# Macrofossil evidence for a rapid and severe Cretaceous–Paleogene mass extinction in Antarctica

**DOI:** 10.1038/ncomms11738

**Published:** 2016-05-26

**Authors:** James D. Witts, Rowan J. Whittle, Paul B. Wignall, J. Alistair Crame, Jane E. Francis, Robert J. Newton, Vanessa C. Bowman

**Affiliations:** 1 School of Earth and Environment, University of Leeds, Leeds LS2 9JT, UK; 2 British Antarctic Survey, High Cross, Madingley Road, Cambridge CB3 OET, UK

## Abstract

Debate continues about the nature of the Cretaceous–Paleogene (K–Pg) mass extinction event. An abrupt crisis triggered by a bolide impact contrasts with ideas of a more gradual extinction involving flood volcanism or climatic changes. Evidence from high latitudes has also been used to suggest that the severity of the extinction decreased from low latitudes towards the poles. Here we present a record of the K–Pg extinction based on extensive assemblages of marine macrofossils (primarily new data from benthic molluscs) from a highly expanded Cretaceous–Paleogene succession: the López de Bertodano Formation of Seymour Island, Antarctica. We show that the extinction was rapid and severe in Antarctica, with no significant biotic decline during the latest Cretaceous, contrary to previous studies. These data are consistent with a catastrophic driver for the extinction, such as bolide impact, rather than a significant contribution from Deccan Traps volcanism during the late Maastrichtian.

## Abstract (web summary)

Debate surrounds the causes, timing, and effects of the Cretaceous-Paleogene mass extinction, 66 Ma. Here, using new collections of marine macrofossils from Seymour Island, Antarctica, the authors show that the extinction was both rapid and severe in the high southern latitudes, contrary to previous studies.

## Graphical abstract

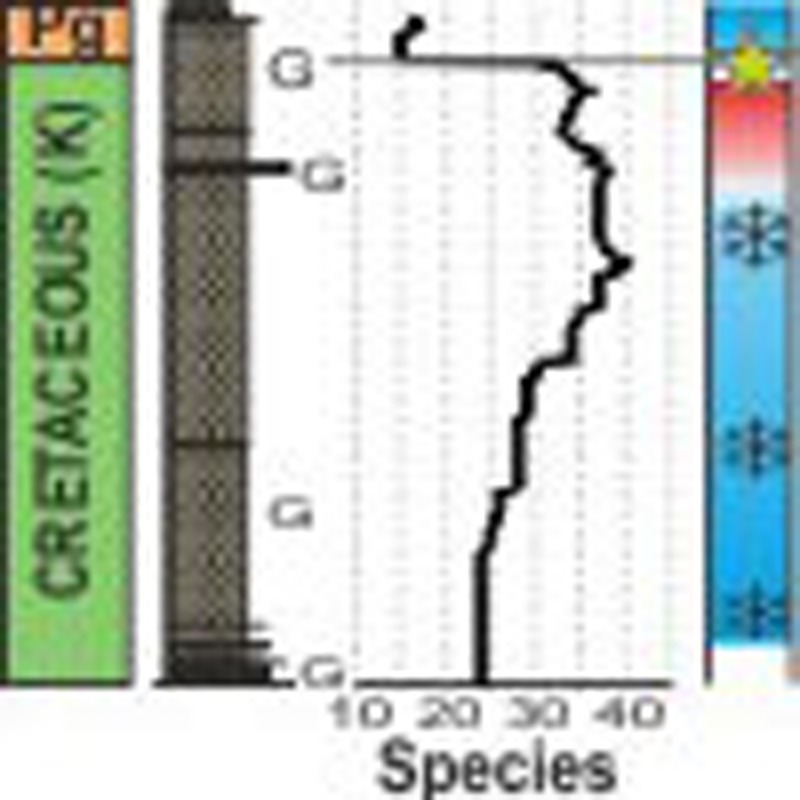

The Cretaceous–Paleogene (K–Pg) mass extinction at 66 Ma is the most intensively studied of the ‘Big Five' crises to have affected life during the Phanerozoic^1^,^2^,^3^,^4^,^5^,^6^,^7^,^8^. The extinction led to a fundamental restructuring of global ecosystems and the rise of modern taxonomic groups^7^,^9^,^10^,^11^. Despite this interest, debate continues as to the duration of the crisis as well as the relative contributions of the bolide impact at Chicxulub^1^,^4^, voluminous eruptions from the Deccan Traps large igneous province^12^,^13^, and dynamic climate instability during the preceding Maastrichtian stage (72.1–66 Ma)^6^,^14^. Parts of this discourse have particularly focused on the Antarctic fossil record. Previous studies on high southern latitude biotas have claimed the extinction to be either a gradual diversity decline^15^,^16^, a series of extinction pulses linked to episodes of Deccan volcanism^17^ or, for ammonites at least, a single rapid event^18^. In addition, it has been suggested that the intensity of the extinction and environmental stress varied with latitude and was related to the proximity of the impact site or the volcanism (both at mid to low latitudes)^19^,^20^. As a result, the high southern latitudes are thought to have weathered the crisis better than lower latitude regions^15^,^21^,^22^.

The López de Bertodano Formation of southern Seymour Island, Antarctica (Fig. 1) represents one of the most highly expanded onshore Maastrichtian–Danian sedimentary successions in the world with ∼1,000 m of sedimentation in ∼4 Myr (refs 17, 23, 24). At a palaeolatitude of ∼65° S during the latest Cretaceous^25^, this succession represents a true high latitude record of events during this critical time period. The succession exposed on Seymour Island is dominated by silty-clays with occasional, thin glauconite-rich sandstone horizons becoming more prevalent in the uppermost 300 m, along with indurated layers formed of early diagenetic concretions and thin bioturbated sands^26^,^27^,^28^. Despite the lithological homogeneity of the succession, several environmental changes have been proposed that have a bearing on marine biodiversity. The depositional environment of the López de Bertodano Formation is broadly transgressive; the lower portion has been interpreted as relatively shallow water, outer estuarine facies^28^,^29^, with a low-energy, marine shelf facies forming the remainder of the succession^27^,^30^.

Here we address these debates on the timing and intensity of Antarctic marine extinctions with a detailed analysis of marine diversity trends during the Maastrichtian to earliest Paleocene ∼70–65.6 Ma (ref. 23) from the López de Bertodano Formation. We evaluate the nature of the K–Pg extinction, its abruptness and intensity in this region. In addition, we test the relationship between palaeoenvironmental changes and diversity, using pyrite petrography as an indicator of palaeoenvironmental conditions. Besides comparisons of existing low-resolution faunal range charts with oxygen isotope data as a proxy for marine palaeotemperature trends^17^, there have been few previous attempts to relate faunal diversity trends in this succession to other local environmental conditions, for example benthic redox changes. We suggest that the K–Pg extinction in Antarctica was as rapid and severe as that seen at lower latitudes, with no evidence for significant precursor extinction events during the latest Maastrichtian, which can be related to the onset of Deccan volcanism or climatic instability.

## Results

### Fossil evidence for diversity and extinction

**Figure 1:**
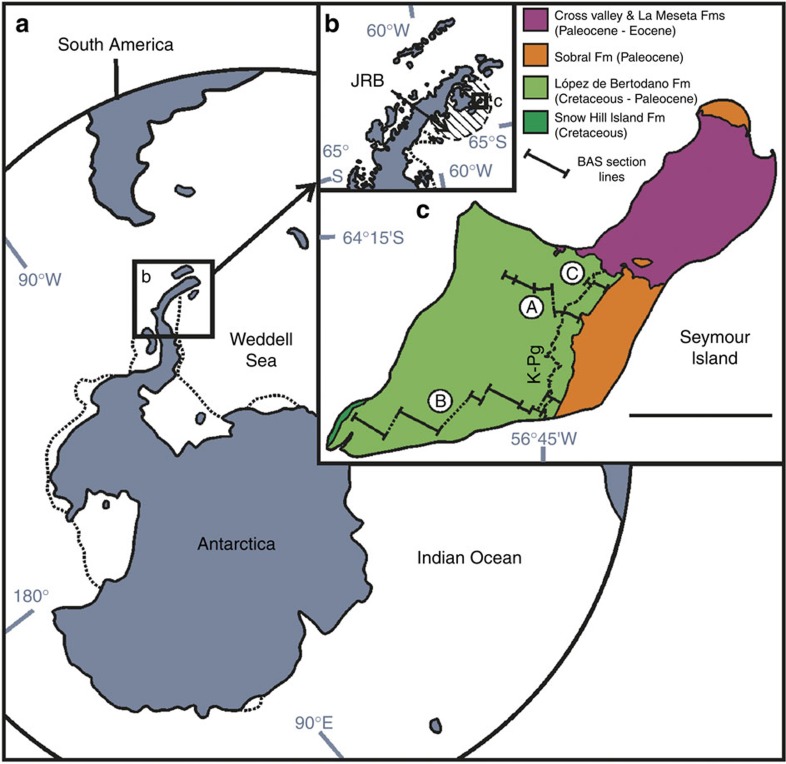
Location map of Seymour Island. (**a**) Modern Southern Hemisphere geography showing location of Antarctica, Antarctic Peninsula highlighted. (**b**) Map of northern Antarctic Peninsula, location of James Ross Basin (JRB) circled and highlighted. (**c**) Geological map of Seymour Island showing locations of principal lithological units, their ages, as well as the locations of British Antarctic Survey section lines mentioned in the text. Location of K–Pg boundary indicated by dotted line on **c**. Circled letters in **c** correspond to individual British Antarctic Survey section lines, A, 1999 field season, B, 2006 field season, C, 2010 field season. Scale bar, 5 km. See Supplementary Figs 1–4 for more details.

**Figure 2:**
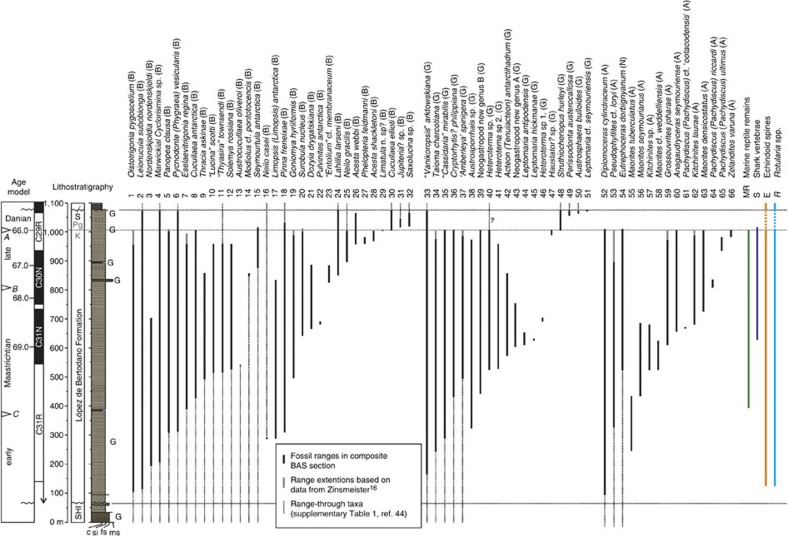
Composite stratigraphic range data for molluscan taxa from the López de Bertodano formation. Range data is plotted against composite lithostratigraphy and age model. Includes new data from this study (see Methods and Supplementary Figs 1–4), and from refs 16, 18 (see also Supplementary Fig. 5). Separated into bivalves (B) (1–32), gastropods (G) (33–51), and cephalopods (A and N) (52–66), with taxa ordered by first appearance in the composite section. Range extensions calculated based on a literature review of molluscan occurrences from underlying formations (Supplementary Table 1), in addition to collections from overlying Paleocene strata^24^,^44^. Also illustrated are the stratigraphic ranges of marine reptile fossils (MR), lamniform shark vertebrae (S), echinoid spines (E) and serpulid worm tubes (*Rotularia* .spp (R) in the composite section. Age model is derived from ref. 23 (see Supplementary Fig. 1 for detailed explanation). Time scale is based on Sr isotope chemostratigraphy (italicized A–C)^68^ and magnetostratigraphy^17^, updated with ages from the Geological Time Scale 2012 (ref. 69) and using the K–Pg boundary datum. G, glauconite-rich intervals. S, Sobral Formation. See Supplementary Note 1 for details of species identifications.

**Figure 3:**
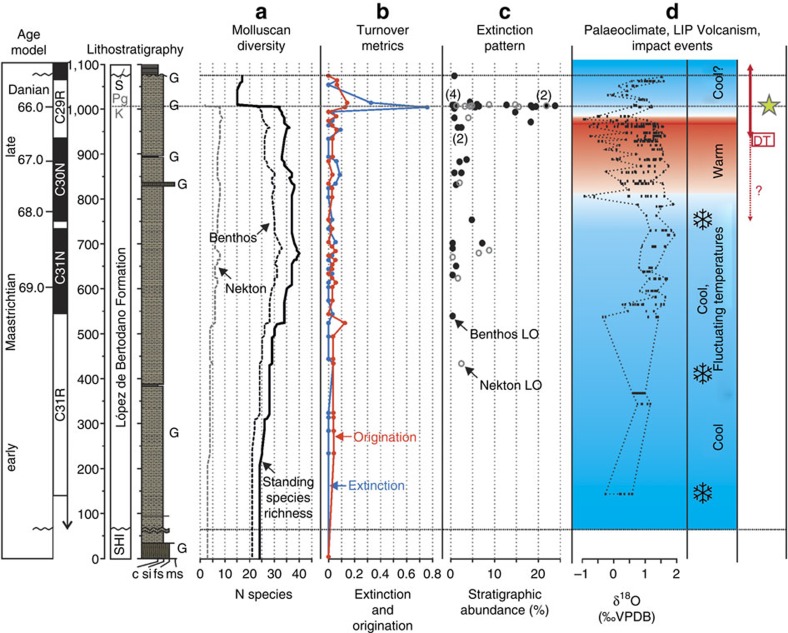
Molluscan diversity and extinction data compared with evidence for Cretaceous–Paleogene palaeoenvironmental change. (**a**) Molluscan diversity for benthos (bivalves and gastropods) and nekton (ammonites and nautiloid) measured as standing species richness in the composite section (Supplementary Data 1), plotted against age model and lithostratigraphy. Composite standing species richness for all molluscan groups plotted as solid black line. Note all nektonic molluscan taxa disappear at the K–Pg boundary (grey dashed line, defined by dinoflagellate cyst biostratigraphy and iridium (Ir) anomaly in paralell section^16^,^30^,^31^. All nekton data species richness and extinction data reanalysed from Witts *et al*.^18^. (**b**) Extinction and origination rates through time based on boundary-crosser methodology^38^, calculated for the entire molluscan fauna (both benthic and nektonic taxa) in 10 m bins (Methods and Supplementary Data 1). See Supplementary Fig. 6 for metrics calculated for benthos and nekton individually. (**c**) Analysis of extinction pattern based on ‘Meldahl's method'^40^,^41^ with last occurrences (LO) in the composite section plotted against stratigraphic abundance S (measured as a %), separated into benthos (bivalves and gastropods) and nekton (ammonites and nautiloid). Numbers in parentheses indicate number of overlapping data points (that is, where taxa have the same value of S). Data are consistent with a single mass extinction event affecting common molluscan taxa (those with a higher value of S) coincident with the K–Pg boundary. (**d**) Palaeoclimate interpretation based on published data from multiple sections on Seymour Island^17^,^23^,^51^,^65^. Snowflake symbols are ‘cold snaps' of Bowman *et al*.^23^, red arrow labelled DT, approximate duration of the main phase of Deccan Trap volcanism (correlated to section and age model using magnetostratigraphy, based on Schoene *et al*.^12^ and Renne *et al*.^13^). Dashed red line and ? illustrates uncertainty surrounding the timing of initial onset of Deccan volcanism^12^,^70^. Yellow star, position of Ir anomaly at the K–Pg boundary in a parallel section^30^ taken as a global marker of the Chicxulub impact event^32^ (see Supplementary Figs 6 and 7 for further information).

The primary data for this study comprises >6,000 benthic molluscan fossils (bivalves and gastropods) from 377 individual sampling stations accurately located within a series of detailed measured sedimentary sections through the López de Bertodano Formation (Fig. 1; Supplementary Figs 1–4). Species range charts based on these collections were combined and compared directly with data from nektonic and nekto-benthic cephalopod molluscs from the same sections^18^ (Fig. 2) and from previous studies of the K–Pg boundary interval on Seymour Island undertaken by Zinsmeister *et al*.^16^ (Supplementary Fig. 6). The K–Pg boundary on Seymour Island occurs in a 2.5–3-m-thick glauconite-rich horizon 1,007.5 m above the base of our composite section, based on biostratigraphic data from marine palynology^24^,^30^,^31^ (Fig. 2; Supplementary Figs 1–5) and the presence in a parallel section of the globally recognized iridium (Ir) anomaly used as a marker for the K–Pg boundary^30^,^32^ (Fig. 3). Besides an abundant and diverse molluscan fauna, other common faunal elements found throughout the Maastrichtian portion of the López de Bertodano Formation include serpulid worm tubes (*Rotularia*), cidaroid echinoid spines, scaphopods, rare solitary corals, decapod crustaceans, marine reptiles, shark vertebrae and fossil wood bored by *Teredolites*^26^,^27^,^33^. Recent work has also revealed the presence of fossil methane seeps, which are periodically developed on the Maastrichtian sea-floor and are characterized by a distinctive benthic molluscan fauna^34^.

On the basis of the combined data sets, a total of 44 species and 39 genera of benthic molluscs occur within the Maastrichtian portion of the López de Bertodano Formation (Fig. 2) along with 15 species and 9 genera of nektonic and nekto-benthic cephalopods^18^. Focusing on the benthos, a total of 25 out of 37 benthic molluscan species (67%) have their last occurrence at or below the K–Pg boundary and 15 out of 35 benthic molluscan genera disappear during the Maastrichtian (43%) (excluding those represented by a single occurrence: 7 species and 4 genera, respectively). Standing species richness based on range-through data gives a good approximation of taxonomic diversity, and indicates an overall increase in benthic molluscan diversity up-section from the base of the López de Bertodano Formation, culminating in a maximum of 33 species at 690 m (310 m below the K–Pg boundary in our composite section) (Fig. 3a). Many of the benthic molluscan genera and species present in the López de Bertodano Formation are also known from the underlying Santonian-early Maastrichtian Santa Marta and Snow Hill Island formations^35^,^36^,^37^ (Supplementary Table 1). However, a similar increase in species richness is also seen in the cephalopod fauna during the same interval^18^ (Figs 2 and 3), suggesting that this early Maastrichtian diversity increase represents a true ecological signal. Rates of turnover (extinction and origination^38^) generally remain low throughout the Maastrichtian (Fig. 3b) for benthic molluscs in particular (Supplementary Fig. 7), with two periods of slightly elevated extinction rates in the benthos between 850 and 890 m, and at 960 m.

Standing species richness of bivalve and gastropod faunas remains stable until a level ∼40 m below the K–Pg interval (Figs 2 and 3). Above this horizon, there is a slight decline due to the apparent loss of six species, before a significant extinction event between 1,000 and 1,007.5 m in our composite section (that is, the interval directly beneath the K–Pg boundary) where 11 benthic species disappear along with all eight remaining species and genera of cephalopods^18^ (Fig. 2; Supplementary Fig. 7). Turnover metrics clearly indicate a significant peak in extinction rate at this level, which is apparent in both a combined analysis (Fig. 3a,b) and when data are separated into benthos and nekton (Supplementary Fig. 7). Two bivalve species (*Panopea clausa* and *Seymourtula antarctica*) range into the glauconite-rich beds in the earliest Paleocene before disappearing, and based on existing data from Zinsmeister *et al*.^16^, another species (*Surobula nucleus*) also shows this pattern (Supplementary Fig. 6). These taxa are classed as additional victims of the extinction (Fig. 2). Thus combined raw stratigraphic range data suggest the benthic molluscan community at Seymour Island saw a species-level extinction of 56% (14 out of 25 species) at the K–Pg event. Local generic level extinction was lower at only 30% (9 out of 30 genera), although several genera (*Cryptorhytis*, *Haustator*) were extirpated in Antarctica but may have survived the K–Pg event at lower latitudes^10^.

These are also likely to be conservative estimates because many of the taxa that disappear within the Maastrichtian are rare and could therefore be under-sampled with respect to their true stratigraphic range (that is, the ‘Signor-Lipps' effect)^39^. To examine this phenomenon, we first employed a modified version of Meldahl's method^40^,^41^ to our combined records (see Methods). Significantly, the majority of the most common molluscs (those most likely to give a true picture of their stratigraphic range^40^) disappear immediately below the K–Pg boundary (Fig. 3c). This suggests that the preceding decline is likely due to the apparent loss of rare taxa, known as ‘backward smearing'^39^,^40^,^41^. Application of confidence intervals to raw range and occurrence data in the two longest stratigraphic sections (A and B in Fig. 1c and Supplementary Fig. 1) in the form of range extensions^42^ support this assertion, showing a cluster of both 50 and 95% confidence intervals around the K–Pg boundary interval outlined above (Supplementary Figs 2 and 3). Only two benthic taxa with a stratigraphic abundance >10 (*Austroaporrhais* sp. and *Eselaevitrigonia regina*) disappear >10 m below the K–Pg boundary, and only one of these disappearances is close to the interval identified by Tobin *et al*.^17^ as containing a supposed precursor extinction event (see Supplementary Data 1 for more details). Furthermore, extension of their 95% confidence intervals into the K–Pg interval in the two BAS section lines suggests that both these taxa, plus a further rare species of bivalve (*Dozyia drygalskiana*), could be additional victims of extinction in the boundary interval. Assuming this is the case would raise the species-level extinction to 61% (17 out of 28) and generic-level extinction to 36% (12 out of 33).

Directly above the K–Pg boundary a low-diversity molluscan assemblage occurs, dominated by large numbers of the bivalves *Lahillia larseni* and *Cucullaea ellioti*, along with the gastropod *Struthiochenopus hurleyi*^27^,^43^. Beds immediately above the Ir anomaly level also contain a large number of diagenetic concretions containing articulated and disarticulated fish remains^16^, the only such horizon in the Seymour Island succession. A total of eight benthic molluscan species range through the K–Pg boundary and occur in the 60-m-thick Paleocene portion of the López de Bertodano Formation. This number increases to 11 when taxa that disappear in the Maastrichtian but reappear in the overlying Paleocene Sobral Formation are included^43^,^44^ (Figs 2 and 3; Supplementary Data 1). Origination rates peak in the interval containing the extinction horizon and directly above in the earliest Paleocene, and seven benthic molluscan species make their first appearances in the 60-m between the K–Pg boundary and the base of the Sobral Formation, an interval that represents ∼350 kyr (refs 17, 23, 24) with only one of these taxa disappearing during the same interval (Figs 2 and 3). However, diversity of the molluscan fauna fails to recover to levels attained during the Maastrichtian.

### Benthic environmental conditions from pyrite petrography

**Figure 4:**
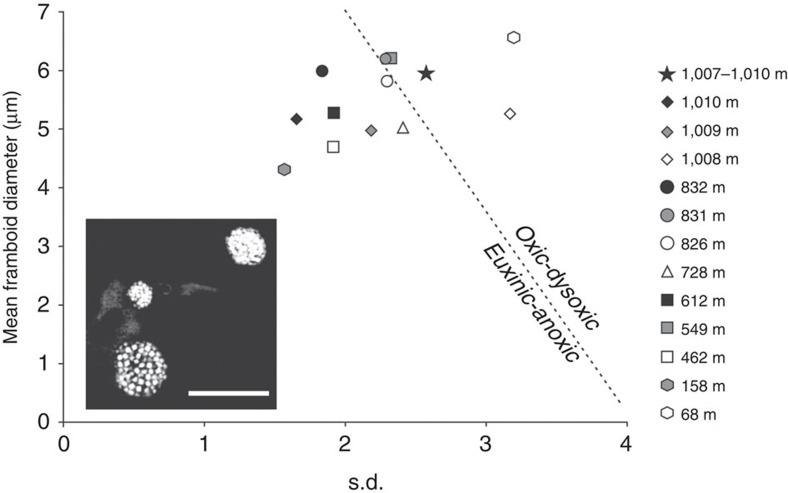
Mean versus s.d. plot of framboid populations from the López de Bertodano Formation. Samples ordered stratigraphically in composite section. Dashed line separates redox conditions and is based on modern calibration^46^. Samples plot in both oxic–dysoxic and anoxic–euxinic fields, indicating rapid redox fluctuations. Inset represents pyrite framboids from sample D5.481.2 (158 m). Scale bar, 10 μm. See Supplementary Fig. 8 and Supplementary Table 2 for more detailed information.

Pyrite petrography was undertaken according to the established procedures^45^. This revealed highly variable pyrite abundance with framboids either being absent (in 10 samples) or present in abundance (in 13 samples). When present, the framboid populations have small mean diameters varying between 4 and 7 μm, and often a considerable ‘tail' of large examples (Supplementary Fig. 8; Supplementary Table 2); this size range is typical of dysoxic (low oxygen) environments in modern settings^46^. Samples with abundant framboid populations are more common in the lower part of the López de Bertodano Formation. Several samples, from closer to the base of the formation, have a narrow size distribution of framboids (minimum diameter 1.5 μm, maximum diameter 14 μm) and a smaller s.d. (1.5–2), more typical of modern anoxic–euxinic environments^46^ (Fig. 4). It is notable that samples from both of the glauconite-rich sandstone units examined in the petrographic analysis, including the interval that yields articulated fish remains immediately above the K–Pg boundary, also have a framboid population typical of rapid fluctuations between dysoxic and anoxic conditions. However, samples from the latest Maastrichtian strata and those above the glauconite-rich interval in the earliest Danian do not contain framboids, possibly indicating that fully oxygenated conditions prevailed on the sea-floor both before and after the K–Pg boundary.

## Discussion

In contrast to all previous studies on the molluscan fauna from Seymour Island^15^,^17^,^30^, our results identify a single, severe K–Pg extinction event in Antarctica affecting both benthic and nektonic taxa, without precursor extinction events. In contrast, Tobin *et al*.^17^ argued for two extinction events, one at the K–Pg boundary itself, and an earlier extinction 40 m below (∼300 kyr prior to the K–Pg boundary), which primarily affected benthic taxa. Tobin and colleagues argued that the earlier crisis was related to a phase of climate warming that has been linked to the onset of Deccan Traps eruptions prior to the K–Pg boundary^47^,^48^. However, there remains significant uncertainty in the precise timing of the main phase of Deccan eruptions relative to the K–Pg extinction^12^,^13^, as well as the magnitude and potential for global environmental change driven by this volcanism^49^, and even the possibility of a cause-and-effect relationship between Deccan eruptions and the Chicxulub impact event^50^. In addition, marine and terrestrial proxy data indicate evidence for regional climate warming in the Antarctic Peninsula region commencing up to 2 million years prior to the K–Pg boundary^23^,^51^ (Fig. 3d). It seems unlikely that any late Maastrichtian warming event significantly stressed the marine fauna. Throughout this interval of warming temperatures, macrofaunal diversity in our composite section remained stable, and extinction rates were low in both the benthic and nekton/nekto-benthic molluscan faunas (Figs 2 and 3; Supplementary Fig. 7). The difference in findings compared with previous studies^15^,^17^ can be explained by our much expanded fossil data set from Seymour Island, which has extended stratigraphic ranges through both the Maastrichtian and the Early Paleocene, and the addition of updated taxonomic information, in particular for benthic molluscs^44^,^52^ (Supplementary Note 1).

The majority of macrofaunal last occurrences are confined to a short interval directly beneath the K–Pg boundary where previously common benthic, and all nektonic molluscs disappear alongside evidence for severe losses in the wider marine community (Figs 2 and 3). For example, remains of large marine vertebrates (marine reptiles, lamniform sharks) are present until the extinction interval, with marine reptiles disappearing only 1 m beneath the K–Pg boundary on Seymour Island^16^,^53^. Providing further evidence for a major benthic disruption, serpulid worm tubes (*Rotularia* spp.) and cidaroid echinoid spines, the most common of all Maastrichtian benthic fossils on Seymour Island, temporarily disappear from the succession at the K–Pg boundary^16^ (Supplementary Fig. 6), only reappearing in the basal levels of the Sobral Formation some ∼350 kyr later (Fig. 2). The record from microfossils is more equivocal. In common with low latitude sections, dinoflagellate cysts exhibit a turnover of taxa at the K–Pg boundary on Seymour Island, but show no significant extinction events^24^,^30^,^31^, as do diatoms^54^. This may be due to the ability to encyst (dinoflagellates) or create resting spores (diatoms) during periods of environmental stress^55^,^56^. Cretaceous planktonic and benthic foraminifera also disappear beneath the K–Pg boundary following a long period of apparent faunal stability in the López de Bertodano Formation^57^. Silicoflagellate assemblages show a similar pattern of stasis followed by an abrupt turnover coincident with the K–Pg boundary on Seymour Island^54^.

The 30–43% extinction of bivalves and gastropods at generic level on Seymour Island is similar to that seen in other Southern Hemisphere molluscan faunas (for example, 21.7–32.1% in Patagonia^10^) These values are also comparable to extinction estimates from Northern Hemisphere K–Pg boundary sections (for example, 30.5% for bivalves and gastropods in the clastic facies of the Gulf Coast, USA^10^,^58^, and 22.5% for bivalves in the chalk facies of Stevns Klint, Denmark^10^,^59^). Estimates for the magnitude of the extinction are similar despite significant differences in the diversity (in terms of generic richness) of these different benthic molluscan faunas from different palaeolatitudes^10^,^44^,^58^,^59^,^60^. Our data are thus also in broad agreement with the suggestion made using global databases, albeit analysed at stage level, that there was no latitudinal variation to the K–Pg losses in these groups^60^. A latitudinal diversity gradient provided no protection against mass extinction for molluscs at the K–Pg boundary, with the polar regions seemingly no safer than the tropics.

Previous studies have postulated that longer-term biotic changes recorded in the James Ross Basin, such as changes in molluscan faunas during the Campanian–Maastrichtian interval, were ‘harbingers' of the K–Pg extinction itself^15^,^30^,^61^. We infer that these changes are more likely recording high-latitude biotic response to environmental perturbations on a longer time-scale, such as the long-term global cooling trend evident at this time^18^,^62^,^63^, and are thus independent of events at the K–Pg boundary.

Nevertheless, existing geochemical, palaeontological and sedimentological data sets from Seymour Island support ideas of a relationship between local palaeoenvironmental changes and marine diversity during the Maastrichtian–Paleocene interval in Antarctica. The lower part of the succession on Seymour Island contains evidence for predominantly cool ocean temperatures during the early Maastrichtian^17^,^23^,^64^ (Fig. 2; Supplementary Fig. 7) supporting records from lower latitudes^62^,^63^. Although lithological homogeneity and lack of key horizons makes a detailed analysis of water depth changes difficult, the shallow water setting proposed for this interval^26^,^28^,^29^, correlates to a period of low molluscan diversity in our sections (Figs 2 and 3; Supplementary Fig. 7). A transition to a more offshore environment occurred during the mid Maastrichtian^26^,^28^,^29^, under a variable temperate climate with fluctuating temperatures and occasional ‘cold-snaps' when sea ice may have developed on the Antarctic margin^17^,^23^,^51^. This interval is linked to an increase in diversity and establishment of a more diverse and stable benthic and nektonic molluscan fauna (Figs 2 and 3). Warming in the late Maastrichtian occurred alongside a slight facies change, with more frequent deposition of glauconite-rich horizons perhaps indicative of subtle changes in water depth and the nature of the substrate^26^. This may account for the minor increase in benthic extinction rates recorded in this interval (Supplementary Fig. 7). Warming is followed by further cooling in the latest Maastrichtian^17^,^24^,^51^,^65^, and evidence for an overall shallowing of the basin into the Paleocene^26^,^29^,^30^. The cluster of last occurrences at the K–Pg boundary appear unrelated to significant facies change.

Integration of our new pyrite petrographic analysis with these data reveals a broadly inverse relationship between diversity in the López de Bertodano Formation and the occurrence of framboid-rich samples. This suggests that, contrary to previous studies of the López de Bertodano Formation that assumed fully oxic conditions throughout deposition^16^,^26^,^29^, fluctuating redox conditions on the Maastrichtian sea-floor may have been a further factor influencing benthic diversity.

Initial low molluscan diversity during the early Maastrichtian also corresponded with evidence for the periodic development of shallow water dysoxia, anoxia and even euxinia (Fig. 4; Supplementary Fig. 8). Significantly, the apparent reappearance of a large number of range-through taxa from underlying formations (Supplementary Table 1) in the mid-López de Bertodano Formation indicates that early Maastrichtian environmental fluctuations were not accompanied by any significant extinction events, at least in the benthos, and any reduction in diversity was temporary (Figs 2 and 3). Oxygenation levels and benthic diversity subsequently improved, although framboid evidence for redox conditions fluctuating between dysoxia and anoxia–euxinia is present in samples in at least three distinct levels in the upper López de Bertodano Formation (Fig. 4; Supplementary Fig. 8).

Interestingly, these include the glauconite-rich sandstone horizons immediately above the K–Pg boundary, suggesting the development of local bottom-water anoxia in the aftermath of the extinction. Although the occurrence of similar conditions associated with another, older, glauconite-rich horizon in the Maastrichtian suggest this is not unusual (Supplementary Fig. 8), certain characteristics of the extinction interval suggest the development of harsh conditions in the water column as well as on the sea-floor at this time. These include the loss of pelagic and nektonic/nektobenthic macrofauna^18^,^53^ (Fig. 2), disruption to planktonic microfauna and flora^31^,^54^,^57^, and occurrence of large numbers of articulated fish remains immediately above the K–Pg boundary on Seymour Island^16^. The origin of recurrent ‘fish-kill' events in this short stratigraphic interval is unclear; Zinsmeister^16^ suggested they may have resulted from large-scale algal or bacterial blooms, or unspecified changes in water chemistry following the end-Cretaceous impact event. Such blooms of opportunistic primary producers and geographically heterogeneous high-stress marine conditions have been hypothesized worldwide in the aftermath of the Chicxulub impact^66^, following perturbation or collapse of the global marine food web due to mass extinction of many groups of phytoplankton^4^. In Antarctica, local benthic anoxia likely led to enhanced preservation potential for victims of the subsequent ‘fish-kills' by limiting the activity of benthic scavengers. The loss of common scavenging taxa such as many epifaunal gastropods and echinoids in the aftermath of the extinction event on Seymour Island (Fig. 2), may also have favoured enhanced preservation of fish carcasses. The benthos of this immediate post-extinction interval is also characterized by unusual high abundance ‘blooms' of presumably opportunistic molluscan taxa, primarily represented by the infaunal bivalve *Lahillia larseni* and the aporrhaid gastropod *Struthiochenopus hurleyi*^27^,^43^.

Pyrite framboid data probably indicate a return to oxygenated sea-floor conditions above the boundary interval (Supplementary Fig. 8). However, successive acmes of several species of dinoflagellate cyst^31^, and an increase in diatom resting spores^54^ in the 60 m stratigraphic interval above the K–Pg boundary may indicate the persistence of unstable marine conditions and a perturbed marine ecosystem following the extinction on Seymour Island. Despite a peak in origination rates at and above the K–Pg interval (Fig. 3), significant recovery of benthic species richness only commenced, ∼350 kyr after the extinction, just prior to initial deposition of the Sobral Formation^24^,^44^. This provides some support for the hypothesized continuation of unusual environmental conditions into the early Danian.

In summary, intensive collecting of marine fossils from the Maastrichtian-earliest Paleocene López de Bertodano Formation on Seymour Island, Antarctica, reveals that, contrary to all previous studies, there was a single, abrupt extinction at the end of the Cretaceous at this location. We find no counter-evidence for any precursor biotic crises at this high southern palaeolatitude. Our results support the idea that a sudden event such as the Chicxulub bolide impact and associated rapid environmental deterioration^4^,^22^,^32^ was the most probable cause of the K–Pg mass extinction. Although recent dating estimates for onset of the main phase of the Deccan Traps large igneous province do suggest a complex temporal coincidence between volcanism and impact at this time^12^,^13^, the precise environmental effects of this volcanism on global ecosystems remain unclear^49^.

Prior to the K–Pg extinction interval, minor diversity fluctuations in Antarctica were linked to local water depth changes with associated changes in seawater temperature. Pyrite framboid data indicates fluctuations in local benthic oxygenation levels may also have played a role in controlling diversity of the marine fauna. In conjunction with the temporary absence of scavenging benthic taxa in the immediate aftermath of the mass extinction event, this provided favourable conditions for fossilization of well-preserved fish directly above the K–Pg boundary in Antarctica, perhaps related to transient unstable marine conditions following the Chicxulub impact. Although evidence for rapid climate oscillations and a late Maastrichtian warming event are present on Seymour Island^17^,^23^,^51^, they are not associated with any significant diversity decline in the marine macrofauna prior to the K–Pg boundary. This argues against models that invoke rapid Maastrichtian climate changes as a significant stressor on pre-extinction communities^6^,^14^. The losses among the benthic invertebrates in Antarctica due to the K–Pg extinction are closely comparable with those recorded from lower latitudes, despite the overall lower diversity of the Antarctic molluscan fauna, and do not support the theory of latitudinal extinction selectivity during this major mass extinction event^22^,^60^.

## Methods

### Sampling strategy

Our primary data are derived from extensive macrofossil collecting in three sedimentary section lines spanning the López de Bertodano Formation and K–Pg interval. All sub-sections that comprise the composite section were measured perpendicular to strike using a Jacob's staff and tape measure. Fieldwork was undertaken during three field seasons to Seymour Island and encompassed the main outcrop of the López de Bertodano Formation in the southern part of the island^24^,^27^,^44^,^51^. The island is ice-free and exposure excellent. Sections DJ.959, DJ.957, DJ.952 and DJ.953 were made during the 1999 field season, and are located close to the central portion of the outcrop, commencing in the mid-levels of the López de Bertodano Formation representing the informal mapping units Klb7–9 and Ktplb10 of Macellari^26^ (Fig. 1). Section D5.251 (comprising sub-sections D5.212, D5.215, D5.218, D5.219, D5.220, D5.222 and D5.229) was measured and sampled during the 2006 field season and runs perpendicular to strike and approximately parallel to the southern coast of the island, beginning within the uppermost levels of the Snow Hill Island Formation. Sections D9.205, D9.206 and D9.207 were located at the northern end of the outcrop during the 2010 field season, and begin immediately below the K–Pg boundary. All three composite sections extend through the K–Pg boundary and the informal mapping unit Ktplb10 to the unconformable contact with the overlying Sobral Formation (Supplementary Figs 1–4).

Macrofossil collections were made systematically at varying scales during the different field seasons, with sample bins ranging on average from 1 m to intervals 10–15 m thick (see Supplementary Figs 2–4 and Supplementary Data 1 for illustration of sampling intervals and bin length in each individual section line). Changes in the size of sample bins within and between individual section lines were necessary during field collecting due to the nature of the ‘scarp and dip-slope' topography that predominates on southern Seymour Island, whereby fossils are invariably more common on dip-slopes than scarps. Collections were made at each station until a representative collection of all the obvious macrofossil types had been obtained; just as it was not possible to standardize sample bin size, so it was not possible to use a standard collecting time either. For these reasons we chose to focus only on range-through data and standing species richness to estimate changes in taxonomic diversity across the K–Pg boundary. In Supplementary Fig. 7, we have plotted variations in sample species richness through all three of our sections, including data from Zinsmeister^16^ (Supplementary Fig. 6). These all show fairly regular variation around a sample mean, but no major trends that could be linked to any obvious form of either local or global environmental variation. Such small-scale fluctuations in species richness are an inevitable consequence of specimen collection in a scarp and dip-slope terrain, and are unlikely to represent any true response to environmental change.

Correlation between section lines and the construction of a composite section was achieved using several stratigraphic tie-points, notably the glauconite-rich beds that mark the K–Pg boundary and a further prominent glauconite-rich horizon present in all section lines ∼174 m below the K–Pg. To enable a full analysis of extinction patterns at the K–Pg boundary, field data derived from British Antarctic Survey sampling was also supplemented with additional data from Zinsmeister^16^ (Supplementary Fig. 6). Zinsmeister's^16^ macrofossil collections were taken from a series of short (20 m) sections measured and sampled during a detailed along-strike mapping study of the K–Pg boundary across ∼5.5 km of southern Seymour Island. When plotting these additional fossil occurrences the base of the ‘Lower Glauconite' horizon of Zinsmeister^16^ is taken as a reference plane, and assumed to be equivalent to the base of the glauconite-rich beds and K–Pg boundary in our composite measured section at a stratigraphic height of 1,007.5 m (refs 31, 51). For all these stratigraphic correlations, we assume planar bedding along strike. The unconformable contact at the base of the Sobral Formation is also useful as a tie-point, although it can be demonstrated that on a regional scale the degree of erosion of the upper levels of the López de Bertodano Formation changes subtly along strike across the island^26^,^67^.

The occurrence of glauconite-rich horizons, such as those that mark the K–Pg interval, suggest periods of slower, condensed sedimentation. The base of these units appears gradational in the field^16^,^24^,^27^,^30^ and high-resolution palynological studies^18^,^23^,^24^ show they are not associated with significant sedimentary hiatuses in the studied sections, but probably represent conformable facies boundaries^24^.

### Fossil data analysis

Over 6,000 fossils of benthic molluscs (bivalves and gastropods) were examined during this study, with 5,710 identified to at least generic level, these have been combined with >700 cephalopod macrofossils^18^ for an examination of overall diversity of the molluscan fauna. Following taxonomic identification and reassessment (Supplementary Note 1), first and last occurrence data from individual section lines were used to construct a composite range chart using the stratigraphic tie points outlined above. Changes in stratigraphic bin size were accounted for by taking the base of the stratigraphic bin in which a species first occurred as the first appearance, and the top of the stratigraphic bin in which a species last occurred as the last appearance. While this introduces a degree of error into the results (for example, where a sampling bin straddles the K–Pg boundary in a single stratigraphic section), it is negligible given the expanded nature of the succession. A presence–absence data set based on this range data (Supplementary Data 1) was used to calculate standing species richness variations throughout the section, supplemented with additional collections from the overlying Paleocene Sobral Formation^24^,^44^ and a literature review to identify range-through taxa from older, underlying formations (Supplementary Table 1). To assess changing rates of biotic turnover through the succession, the presence–absence data set was split into 10 m bins and both extinction (*E*_r_) and origination (*O*_r_) rates calculated for each 10 m bin using the boundary-crosser methodology outlined by Foote^38^:


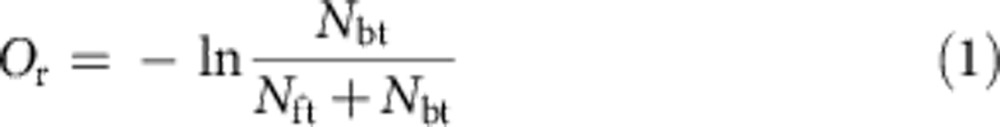



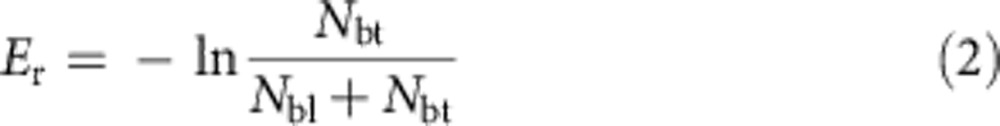


where *N*_bt_=number of range-through taxa, *N*_ft_=number of taxa that originate within any given 10 m bin and cross the top boundary of that bin and *N*_bl_=number of taxa that cross the bottom boundary of the bin but have their last occurrence within the bin. These should be considered as ‘extinction' and ‘origination' rates only in the local context, and are not expressed relative to bin duration. Available evidence suggests overall sedimentation rates remained high throughout deposition of the López de Bertodano Formation at 0.1–0.2 mm per year (ref. 17) (Supplementary Fig. 5) indicating that any variation in bin duration is likely to have a negligible effect on the magnitude of biotic turnover rates.

To test the hypothesis of multiple extinction events and visually assess the pattern of taxonomic turnover more generally through the Maastrichtian, we also employed the stratigraphic abundance method of Meldahl^40^. Stratigraphic abundance (*S*, the percentage of sample intervals in which a given taxon occurs) was calculated using a recently modified method^41^ to enable us to include data from all three studied sedimentary sections in addition to data from Zinsmeister^16^:


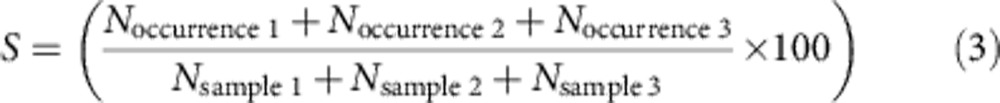


where *N*_occurrence 1_ is the number of occurrences of a given species in section 1, and *N*_sample 1_ is the number of samples in section 1. A plot of *S* versus last occurrence provides a visual estimate of the likely position of an extinction horizon based on the disappearance of the most common taxa in an assemblage (Fig. 3c), and along with a plot of the frequency distribution of last occurrences in a stratigraphic section (Supplementary Fig. 7), can be compared with simulated models of sudden and gradual extinction^40^. We included all molluscan taxa in this analysis, including a reanalysis of the nekton to include additional data from field collections made in 2010 (data reanalysed from ref. 18). To investigate the extent to which the data is influenced by the ‘Signor-Lipps Effect'^39^ due to the sampling strategy, 50 and 95% confidence intervals were calculated for all benthic taxa with >5 occurrences during the Maastrichtian within two BAS section lines (sections A and B, Fig. 1c). We applied the ‘classical' method as summarised by Marshall^42^, and illustrate these confidence intervals as range extensions (Supplementary Figs 3 and 4) using the following equation:


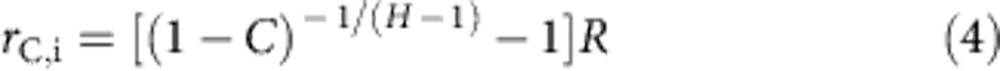


Average gap size between fossil occurrences also provides an unbiased point estimate of the true time of appearance or disappearance in any given stratigraphic section assuming random fossil recovery^42^, and was calculated for the same taxa using:


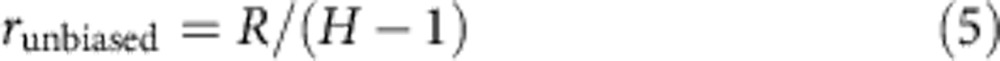


where *r*_C,i_ is the length of the range extension, *r*_unbiased_ is the average gap between fossil occurrences as a percentage of that taxon's stratigraphic range, *C* is the desired confidence level (expressed as a decimal; 0.5 and 0.95), *H* is the number of observed fossil occurrences for a species in an individual section line and *R* is the observed stratigraphic range of the taxon in the same section line. Confidence intervals were only calculated for data from two sections directly sampled by the authors (sections A and B, Fig. 1c), both of which have extended records from the Maastrichtian into the Paleocene. Because of the constraints of the sampling strategy outlined above, confidence intervals were not applied to any composite data set that is derived only from range-through data (Fig. 2).

### Pyrite petrography

Polished blocks were made from 21 bulk sediment samples collected throughout composite section D5.251 (Supplementary Table 2). These were examined using an FEI Quanta 650 scanning electron microscope (SEM) in back-scatter mode to identify microfacies and quantify the diameter of pyrite framboid populations. The size and distribution of pyrite framboids in both ancient and modern sediments are interpreted to result from local redox conditions^45^,^46^. In modern environments, syngenetic framboids form in a narrow iron reduction region developed at the redox boundary, but cease growing in the underlying fully anoxic sulphate reduction zone. Under fully euxinic conditions (where free H_2_S occurs in the water column), syngenetic framboids grow to a maximum diameter of 6–7 μm in the water column before gravity causes them to sink to the seabed^45^. Framboid populations formed under these conditions will exhibit both a small size range and a small s.d. In dysoxic settings, conditions on the seabed are often weakly oxygenated, leading to framboid development in the pore water of the underlying sediments. Here the size range is controlled primarily by the availability of reactants and therefore framboid populations typically grow to larger sizes (up to 20 μm) with a correspondingly higher s.d.^46^. Supplementary Figure 8 presents a ‘Box and Whisker' plot showing the stratigraphic distribution of the sampled horizons and illustration of framboid populations.

### Data availability

The authors declare that all data supporting the findings of this study are available within the article and its Supplementary Information Files.

## Additional information

**How to cite this article:** Witts, J. D. *et al*. Macrofossil evidence for a rapid and severe Cretaceous–Paleogene mass extinction in Antarctica. *Nat. Commun.* 7:11738 doi: 10.1038/ncomms11738 (2016).

## Supplementary Material

Supplementary Information Supplementary Figures 1-8, Supplementary Tables 1-2, Supplementary Note 1 and Supplementary References.

Supplementary Data 1 Raw field data (sheets A-D), composite range, occurrence, and stratigraphic abundance data (sheets E-F), and turnover metrics (origination and extinction) (sheet G).
