# Supplementary material for: Macrofossil evidence for a rapid and severe Cretaceous–Paleogene mass extinction in Antarctica
Source: Nat Commun. 2016 May 26;7:11738. doi: 10.1038/ncomms11738 (PMC4894978; doi:10.1038/ncomms11738)
Supplement: Supplementary Information — Supplementary Figures 1-8, Supplementary Tables 1-2, Supplementary Note 1 and Supplementary References. [file ncomms11738-s1.pdf]

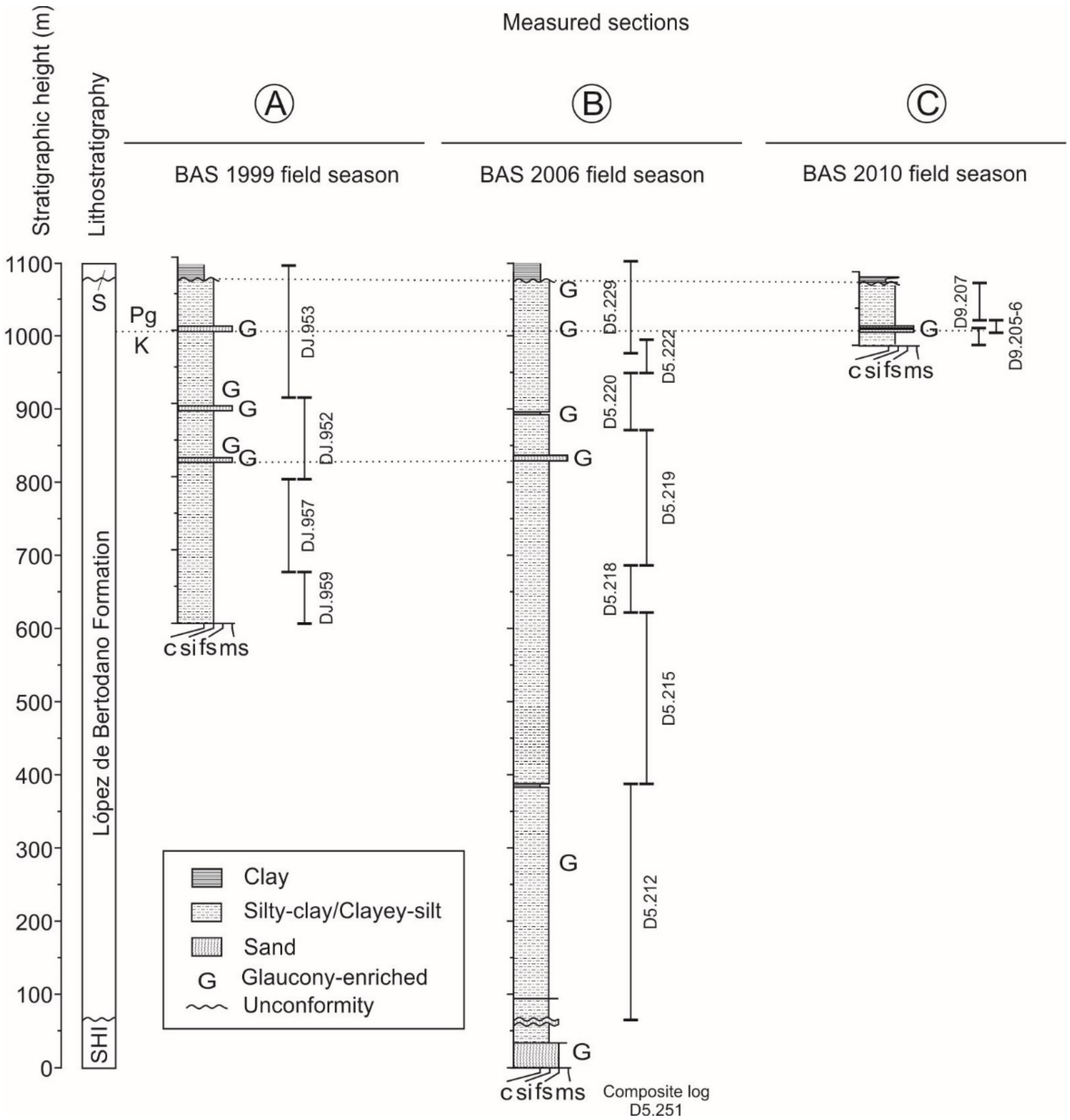

Supplementary Figure 1 | Lithostratigraphy and correlation within the López de Bertodano Formation on Seymour Island. BAS (British Antarctic Survey) sedimentary sections A–C used to construct composite section. Tie points for correlation: K–Pg boundary, unconformable contact with overlying Sobral Formation, and prominent glauconitic horizon 174 m below the K–Pg interval. A–C refer to section identifications used in Main Text Fig. 1. SHI, Snow Hill Island Formation, S, Sobral Formation, K, Cretaceous, Pg, Paleogene. For further details on individual field seasons and section lines see supplementary refs 1–7.

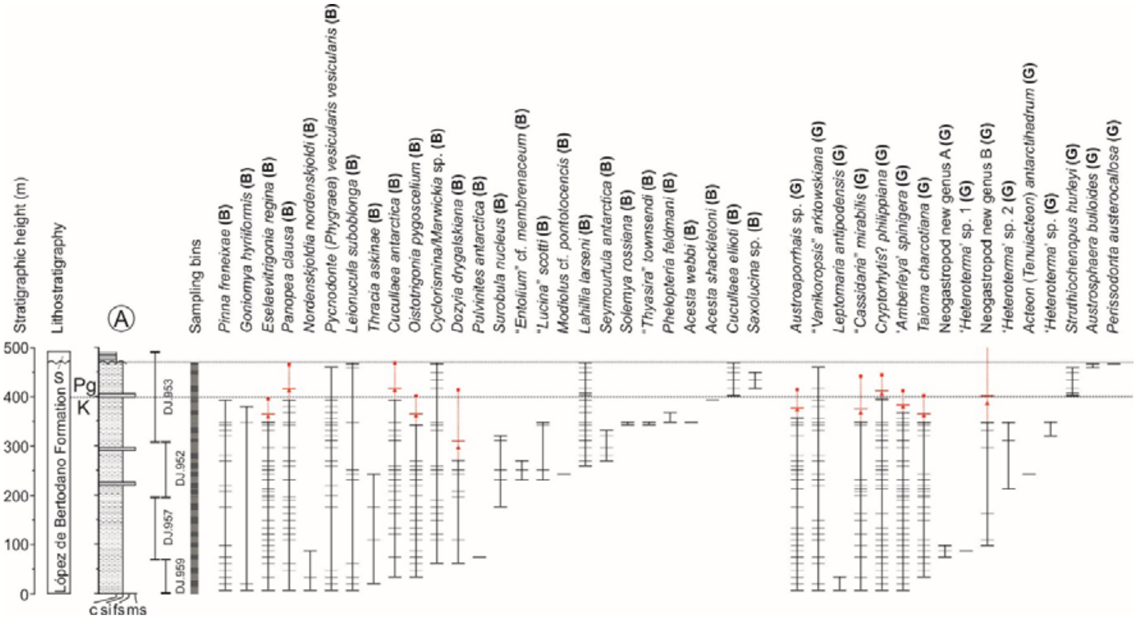

Supplementary Figure 2 | Stratigraphic range and occurrence data for molluscan taxa from the BAS 1999 field season. Lithostratigraphy, sampling bins, and raw stratigraphic range (vertical solid black lines) and occurrence (horizontal black ticks) data for benthic molluscan taxa (bivalves (B) and gastropods (G)) from sub-sections DJ.959, DJ.957, .952, and .953[1,7]. 50% confidence intervals (red triangles), 95% confidence intervals (red squares), and average gap size between occurrences (horizontal red ticks) calculated for Maastrichtian taxa with more than five occurrences. Illustrated as range extensions (vertical red dashed line) calculated using 'classic' confidence interval methodology as summarised by Marshall[8]. See Supplementary Data 1 for raw field data and Supplementary Fig. 1 for lithostratigraphic key.

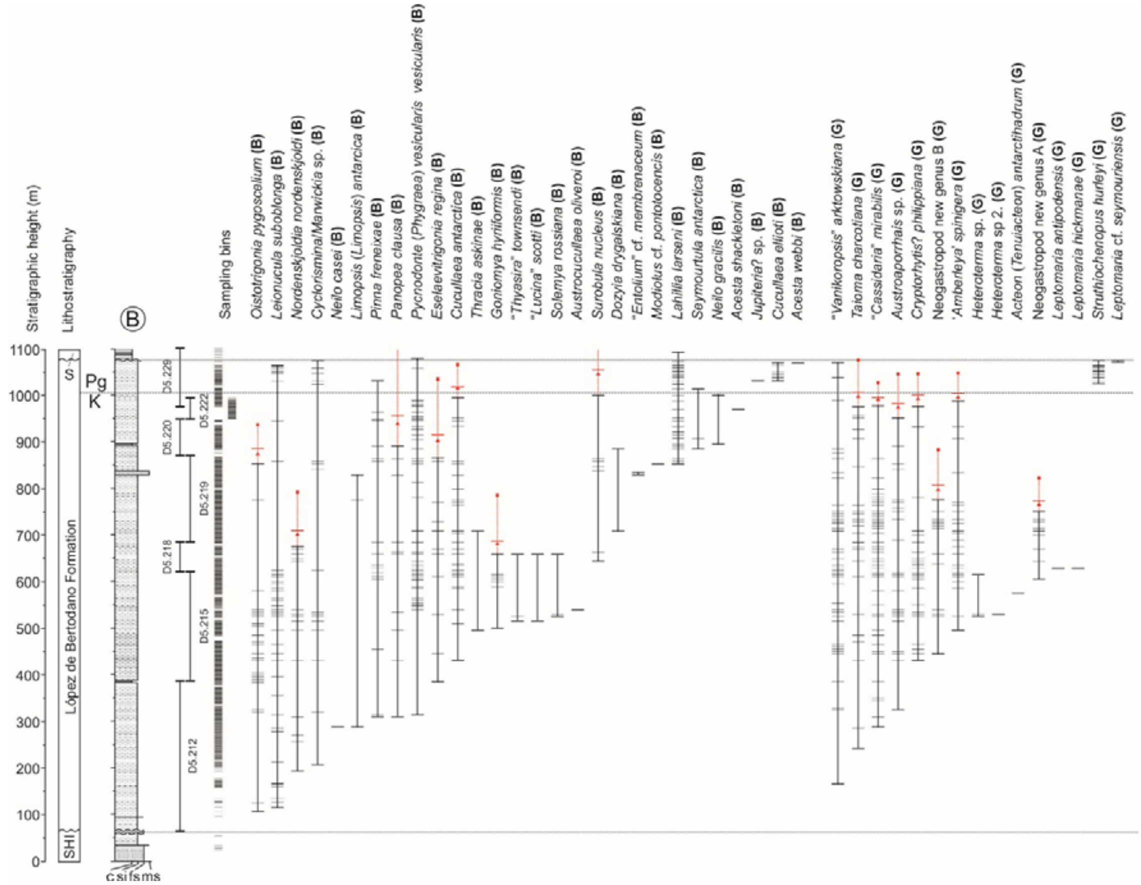

Supplementary Figure 3 | Stratigraphic range and occurrence data for molluscan taxa from the BAS 2006 field season. Lithostratigraphy, sampling bins, and raw stratigraphic range data for benthic molluscan taxa (bivalves (B) and gastropods (G) from sub-sections D5.212, .D5.215, D5.218, D5.219, D5.220, D5.222, D5.229[3–6]. 50% confidence intervals (red triangles), 95% confidence intervals (red squares), and average gap size between occurrences (horizontal red ticks) calculated for Maastrichtian taxa with more than five occurrences. Illustrated as range extensions (vertical red dashed line) calculated using 'classic' confidence interval methodology as summarised by Marshall[8]. See Supplementary Data 1 for raw field data and Supplementary Fig. 1 for lithostratigraphic key.

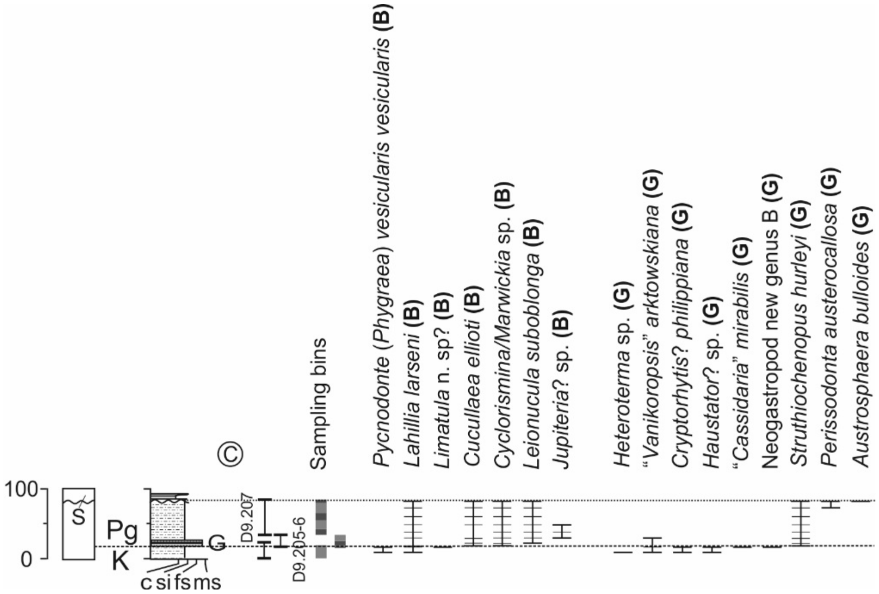

Supplementary Figure 4 | Stratigraphic range and occurrence data for molluscan taxa from the BAS 2010 field season. Lithostratigraphy, sampling bins, and raw stratigraphic range data for benthic molluscan taxa (bivalves (B) and gastropods (G)) from sub-sections D9.205, D9.206, D9.207[2,4,7]. Due to the limited number of Maastrichtian samples, confidence intervals could not be calculated for these sections. See Supplementary Data 1 for raw field data, and Supplementary Figure 1 for lithostratigraphic key.

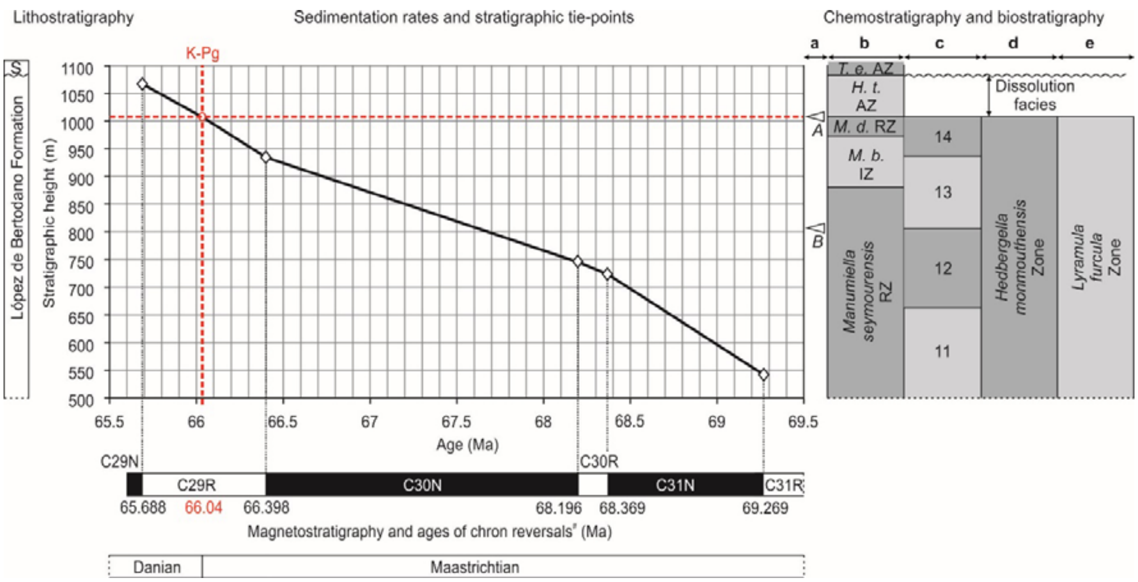

Supplementary Figure 5 | Summary of age model for the López de Bertodano Formation. Magnetostratigraphy taken from Tobin et al.[9] correlated to British Antarctic Survey (BAS) composite section using the K–Pg boundary as a stratigraphic tie-point and assuming planar bedding along strike. # updated chron reversal ages from Gradstein et al.[10] **a**–**e** chemostratigraphy and biostratigraphy. **a**, strontium (Sr) isotope chemostratigraphy[11] arrow A = bivalve *Pycnodonte* cf. *P. vesiculosa*, 0m (relative to K–Pg boundary, $^{87}Sr/^{86}Sr$ = 0.707833. Arrow B = bivalve '*Linotrigonia*' *pygoscelium*, -200m, $^{87}Sr/^{86}Sr$ = 0.707831. Additional $^{87}Sr/^{86}Sr$ data used in age model construction from belemnite *Dimitobelus* (*D.*) *seymouriensis* -636 m[1], $^{87}Sr/^{86}Sr$ = 0.707787. All values confirm a Maastrichtian age when compared to Sr isotope data from lower latitudes[12]. **b**, dinoflagellate cyst biostratigraphy[2–4], *M.b.* IZ = *Manumiella bertodano* Interval Zone, *M. d.* RZ = *Manumiella druggii* Range Zone, *H. t.* AZ = *Hystrichosphaeridium tubiferum* Acme Zone, *T. e.* AZ = *Trithyrodinium evittii* Acme Zone. Paleocene dinoflagellate cyst zones from Antarctica are correlated to New Zealand zones NZDP1 and NZDP2[2]. **c**, ammonite biostratigraphy[5,13], ammonite assemblage zones (11–14) from Olivero[14]. 11, *Maorites tuberculatus*, 12, *Pachydiscus* (*Pachydiscus*) '*ootacodensis*', 13, *Pachydiscus* (*Pachydiscus*) *riccardii*, 14, *Pachydiscus* (*Pachydiscus*) *ultimus*. **d**, foraminiferal biostratigraphy[15]. **e**, silicoflagellate biostratigraphy[16]. Dissolution facies above the K–Pg boundary defined by Huber[15] to account for non-preservation of calcareous microfossils. For further details on the development of this age model see supplementary refs 3–5.

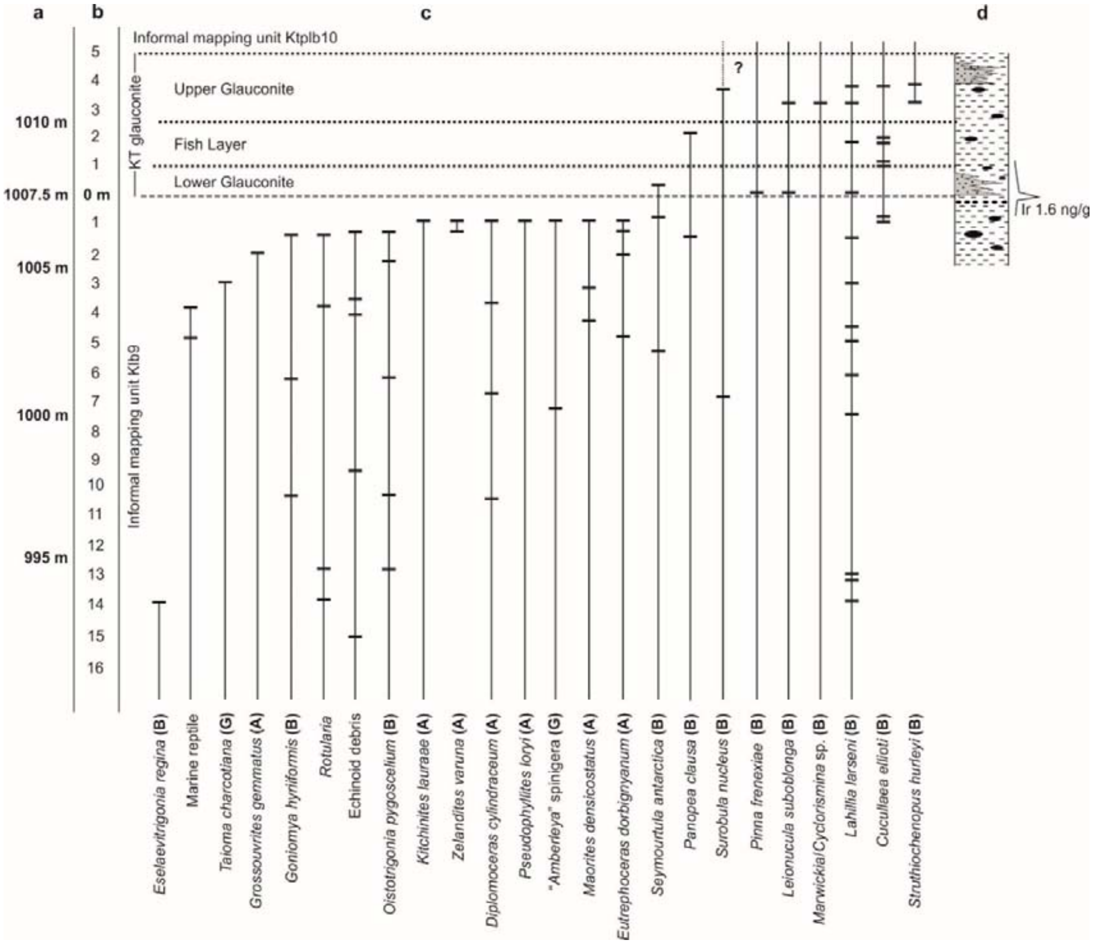

Supplementary Figure 6 | Faunal range and occurrence data, and lithostratigraphy modified from the study of Zinsmeister[17]. Data based on a compilation from 20 short measured section lines across the K–Pg boundary. **a**, correlation between supplementary ref 17 and BAS composite section, assuming base of the "Lower Glauconite" is equivalent to the K–Pg boundary in our composite section (1007.5 m)[2,3,5,6]. **b**, stratigraphic height relative to the base of the "Lower Glauconite"/K–Pg boundary. **c**, stratigraphic nomenclature from supplementary refs 17 and 18, along with faunal range and occurrence data, black lines represent species ranges, horizontal ticks are occurrences and last occurrences in different measured sections, only last occurrences used to extend ranges of taxa in BAS sections. All occurrences used in stratigraphic abundance calculations. Some species names have been changed to bring them into line with revised taxonomy (see supplementary ref 7, Supplementary Note 1 and Supplementary Data 1), (B), bivalve, (G), gastropod, (A), ammonite, '?' next to *Surobula nucleus* illustrates that this species does not range through into the Danian in our composite section. **d**, sedimentary log and lithostratigraphy from supplementary ref 17, iridium (Ir) anomaly in "Lower Glauconite" only from supplementary ref 19.

Supplementary Figure-6B Witts

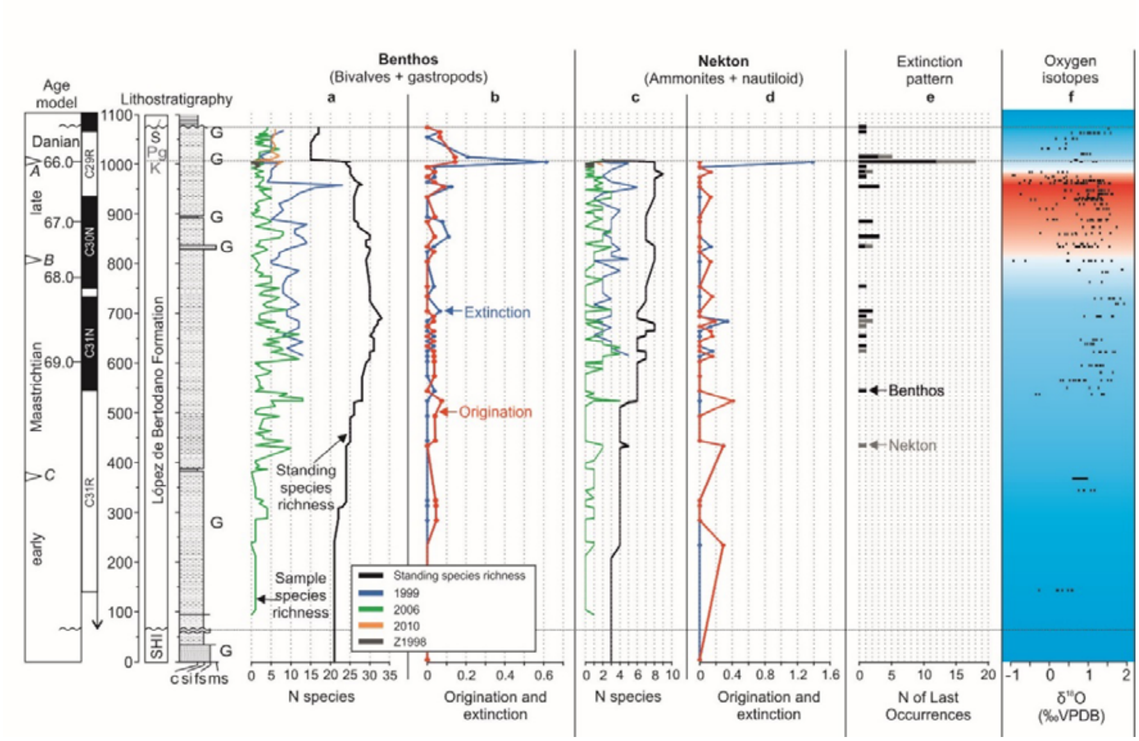

Supplementary Figure 7 | Expanded molluscan diversity and palaeoenvironmental data. Molluscan species richness variations within individual section lines, standing species richness based on composite range-through data (**a**, **c**) and estimated rates of origination and extinction in 10 m binned intervals (**b**, **d**), divided into benthos (**a**, **b**) and nekton (**c**, **d**) (species richness variations in individual sections based on occurrences in Supplementary Figs. 3–6, see Supplementary Data 1). **e**, histogram illustrating pattern of extinction as defined by the number of last occurrences within 10 m binned intervals through the Maastrichtian–Paleocene interval, divided into benthos (black) and nekton (grey). The pattern is consistent with a sudden extinction event at the K–Pg boundary at 1007.5 m, taking into account the Signor-Lipps effect[20]. **f**, raw oxygen isotope data taken from Tobin et al. (supplementary ref 9) and Dutton et al. (supplementary ref 21), used as a proxy for marine palaeotemperature. Blue = cooler overall temperatures, red = warmer overall temperatures, as defined by other palaeoclimate records[4,6]. All data plotted against age model and lithostratigraphy. See Supplementary Figs. 1 and 5 for details and lithostratigraphic key.

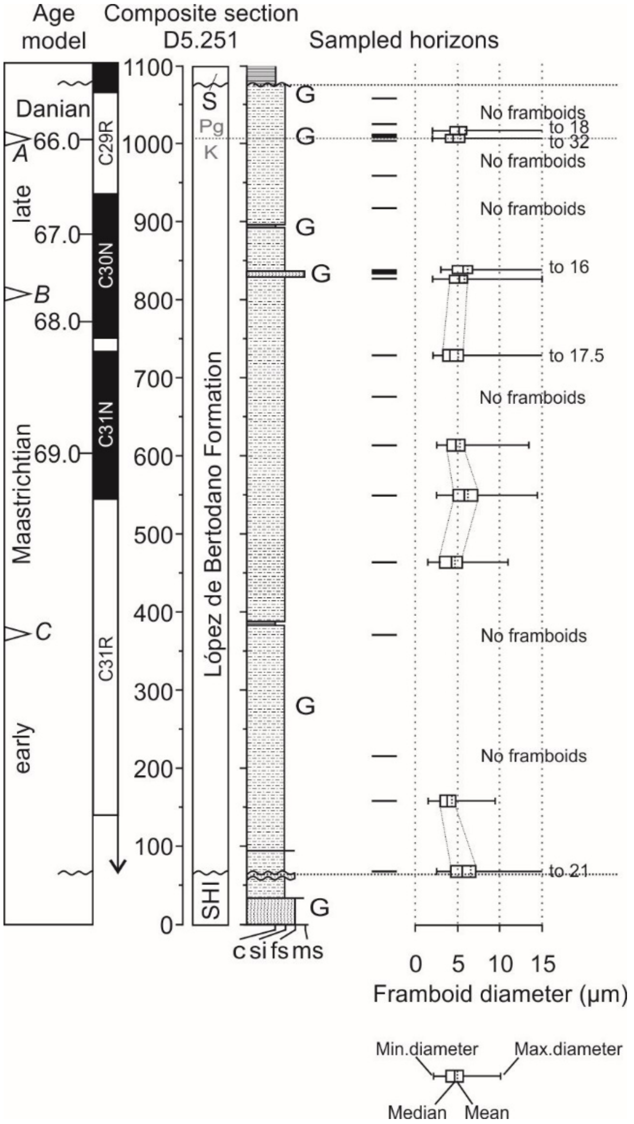

Supplementary Figure 8 | Pyrite framboid data derived from section D5.251 illustrated against lithostratigraphy and age model. Data are illustrated as 'box and whisker' plots[22]. See Supplementary Figs. 1 and 5 for details of age model and lithostratigraphic key.

Supplementary Table 1 | Identification of range-through taxa from Campanian–Maastrichtian strata stratigraphically below the López de Bertodano Formation on Seymour Island.

#	Species *	Location, (Formation, member) ☐	Supplementary reference
1	*Leionucula suboblonga* (B)	Snow Hill Island (SHIF, Karlsen Cliffs Mbr)	25, 26
2	*Solemya rossiana* (B)	Snow Hill Island (SHIF, Karlsen Cliffs Mbr)	25, 26, 27
3	*Modiolus pontotcensis* (B)	Vega Island (SHIF, Cape Lamb Mbr)	25, 28, 29
4	*Nordenskjoldia nordenskjoldi* (B)	Vega Island (SHIF, Cape Lamb Mbr)	29
5	*Austrocucullaea oliveroi* (B)	Vega Island (SHIF, Cape Lamb Mbr)	29
6	*Cucullaea antarctica* (B)	Vega Island (SHIF, Cape Lamb Mbr)	29
7	*Pycnodonte* (*Phygraea*) *vesicularis vesicularis* (B)	Cockburn Island (SHIF, undifferentiated)	30, 31
8	*Seymourtula antarctica* (B)	Cockburn Island (SHIF, undifferentiated)	30, 31
9	*Oistotrigonia pygoscelium* (B)	Vega Island (SHIF, Cape Lamb Mbr)	29
10	*Eselaevitrigonia regina* (B)	Vega Island (SHIF, Cape Lamb Mbr); Snow Hill Island (SHIF, Haslum Crag Mbr)	23, 25, 26, 29
11	"*Lucina*" *scotti* (B)	Snow Hill Island (SHIF, Karlsen Cliffs and Haslum Crag mbrs)	25, 26, 27
12	"*Thyasira*" *townsendi* (B)	Snow Hill Island (SHIF, Karlsen Cliffs and Haslum Crag mbrs)	25, 26, 27
13	*Cyclorismina*/*Marwickia* sp. (B)	Snow Hill Island (SHIF ?Karlsen Cliffs Mbr)	25, 26
14	*Panopea clausa* (B)	Humps Island (SHIF, undifferentiated)	32
15	*Thracia askinae* (B)	Vega Island (SHIF,	25, 28, 29

		Cape Lamb Mbr)	
16	"*Amberleya*" *spinigera* (G)	Ula Point, James Ross Island (SHIF, undifferentiated)	33
17	"*Vanikoropsis*" *arktowskiana* (G)	Snow Hill Island (SHIF, ?Karlsen Cliffs Mbr)	7, 26
18	"*Cassidaria*" *mirabilis* (G)	Snow Hill Island (SHIF, ?Karlsen Cliffs Mbr)	7, 26
19	?*Cryptorhytis phillippiana* (G)	Snow Hill Island (SHIF, ?Karlsen Cliffs Mbr)	7, 26
20	*Taioma charcotiana* (G)	Snow Hill Island (SHIF, ?Karlsen Cliffs Mbr)	7, 26
21	*Pseudophyllites loryi* (A)	Vega Island (SHIF, Cape Lamb Mbr)	10, 11, 29
22	*Diplomoceras cylindraceum* (A)	Vega Island (SHIF, Cape Lamb Mbr), Snow Hill Island (SHIF, Sanctuary Cliffs, Karlsen Cliffs, Haslum Crag mbrs)	10, 11, 23, 34
23	*Eutrephoceras dorbignyanum* (N)	Vega Island (SHIF, Cape Lamb Mbr), James Ross Island (SMF, Lachman Crags Mbr)	10, 29, 35

Key: *(B), bivalve, (G), gastropod, (A), ammonite, (N), nautiloid.

☐ SHIF, Snow Hill Island Formation, SMF, Santa Marta Formation.

For information on the overall lithostratigraphy of the James Ross Basin see supplementary references and references therein[1–7,11,14,23–24].

Supplementary Table 2 | Pyrite framboid data taken from stratigraphic section D5.251.

Sample #	Stratigraphic height in composite section D5.251 (m)	N	Mean	S.D.	Min. F.D.	Max. F.D.
D5.1351.1	1058	N.A.	N.A.	N.A.	N.A.	N.A.
D5.1308.1	1023	N.A.	N.A.	N.A.	N.A.	N.A.
D5.1295.1	1007-1010	105	5.95	2.58	2	18
D5.1296.1	1010	104	5.17	1.65	2.5	13.5
D5.1294.1	1009	103	4.97	2.19	2	12.5
D5.1293.1	1008	103	5.26	3.17	1	31
D5.1292.1	1007	N.A.	N.A.	N.A.	N.A.	N.A.
D5.1290.1	1002	N.A.	N.A.	N.A.	N.A.	N.A.
D5.1237.1	958	N.A.	N.A.	N.A.	N.A.	N.A.
D5.1213.1	916	N.A.	N.A.	N.A.	N.A.	N.A.
D5.1164.1	832	102	5.99	1.84	3	14.5
D5.1163.1	831	99	6.2	2.3	3	16
D5.1160.1	826	104	5.82	2.3	2	15.5
D5.1108.1	728	102	5.02	2.41	2	17.5
D5.1053.1	674	N.A.	N.A.	N.A.	N.A.	N.A.
D5.696.1	612	104	5.27	1.92	2.5	13.5
D5.370.1	549	101	6.21	2.33	2.5	14
D5.985.1	462	104	4.69	1.92	1.5	11
D5.902.1	380	N.A.	N.A.	N.A.	N.A.	N.A.
D5.892.1	370	N.A.	N.A.	N.A.	N.A.	N.A.
D5.535.1	213	N.A.	N.A.	N.A.	N.A.	N.A.
D5.481.2	158	105	4.31	1.57	1.5	9.5
D5.406.1	68	100	6.56	3.2	2.5	21

N, number of framboids measured in sample, Mean, mean framboid diameter, S.D., standard deviation of framboid diameters, Min. F.D., minimum framboid diameter, Max F.D., maximum framboid diameter.

Supplementary Note 1

Classification of benthic molluscan taxa from the Maastrichtian–Danian López de Bertodano Formation and taxonomic remarks, bivalves arranged in taxonomic order according to Bouchet and Rocroi[36] and Bieler et al.[37], and gastropods according to Bouchet and Rocroi[38] and Ponder and Limberg[39]. See also Crame et al.[7], Zinsmeister and Macellari[25], Stilwell et al.[40], and Beu[41] for further discussion and full descriptions of Cretaceous–Paleogene molluscan taxa from Antarctica.

Photographs and information on specimens collected and described by other authors from Seymour Island[7,25,40] currently held in the collections of the Paleontological Research Institute (PRI), Ithaca, NY, USA are available online at: http://www.pricollectionsdatabase.org/

Class: Bivalvia

Leionucula suboblonga[7.25,26,40,41]

Solemya rossiana[27]

Jupiteria? sp.[40,41]

Neilo casei[25,41]

Neilo gracilis[25,41]

Modiolus cf. *pontotocencis*[25]

Nordenskjoldia nordenskjoldi[25]

Austrocucullaea oliveroi[25]

Cucullaea antarctica[25]

Cucullaea ellioti[25,40]

- In the BAS composite section specimens of *Cucullaea ellioti* occur only above the K–Pg boundary, whereas Zinsmeister[17] records several examples from immediately below the Ir-bearing Lower Glauconite horizon.

Limopsis (*Limopsis*) *antarctica*[25,42]

Phelopteria feldmanni[25]

Pulvinites antarctica[25,43]

Pinna frenexiae[25]

Pycnodonte (*Phygraea*) *vesicularis vesicularis*[25,44,45]

- Two distinct species of *Pycnodonte* have been described from Seymour Island by Zinsmeister and Macellari[25], they recognised the high degree of morphological plasticity shown by both. It is likely that all specimens from the López de Bertodano and Sobral Formations are ecomorphs of a single, highly variable species[44], the widespread *Pycnodonte* (*Phygraea*) *vesicularis vesicularis* (see Supplementary ref 45 for discussion).

"*Entolium*" cf. *membranaceum*[25,46–48]

- See supplementary ref 46 for discussion of the current problems associated with pectinid taxonomy. BAS specimens are too poorly preserved to separate into two species as per Zinsmeister and Macellari[22]. "*Entolium*" s.s. is used to describe many smooth Late Cretaceous pectinids, with "*E.*" *membranaceum* often considered a cosmopolitan latest Cretaceous species[47,48].

Limatula sp.[7,40]

- This is the same as the specimens referred to as *Limatula* sp. nov in supplementary ref 7.

Acesta shackletoni[25]

Acesta webbi[25]

Seymourtula antarctica[25]

Oistotrigonia pygoscelium[25]

Eselaevitrigonia regina[25]

"*Lucina*" *scotti*[25,27]

"*Saxolucina*" sp.[40,41]

"*Thyasira*" *townsendi*[25,27]

Dozyia drygalskiana[25]

Lahillia larseni[25,40]

Cyclorismina/*Marwickia* sp.[25,40,41]

- Taxa from Seymour Island assigned to these two genera were separated based on differences in size, shape of the umbo, and presence of anterior lateral tooth in "*Marwickia woodburnei*", but only articulated specimens with no dentition visible have been illustrated[25,40]. The majority of BAS specimens are also articulated, in many cases exhibit rather poor preservation and externally appear identical. We are thus unable to resolve the material in this collection into two distinct genera or species.

Surobula nucleus[25]

Panopea clausa[25]

Goniomya hyriiformis[25]

Thracia askinae[25]

Class: Gastropoda

Leptomaria cf. *seymouriensis*[49]

Leptomaria hickmanae[49]

Leptomaria antipodensis[49]

"*Amberleya*" *spinigera*[7,50]

- *Amberleya* sensu stricto is now restricted to the Jurassic type species[50]. "*A.*" *spinigera* is probably a member of *Ambercyclus* - with morphology intermediate between the genera *Amberleya* and *Eucyclus* [50].

Haustator? sp.

- High-spired turritellid gastropod with flat whorl sides. Overall morphology suggests placement of two worn specimens from the interval beneath the K–Pg boundary in the genus *Haustator*. But the material is too poorly preserved for a definitive identifiation. Turritellid gastropods appear rare in the López de Bertodano and Sobral Formations[40,41].

"*Vanikoropsis*" *arktowskiana*[7]

Austroaporrhais sp.[51]

- Material in BAS collections is too poorly preserved to be separated into the various Maastrichtian species of *Austroaporrhais* previously identified from Seymour Island[51]

Perissodonta austerocallosa[7,41]

Struthiochenopus hurleyi[40,51]

Neogastropod new genus A[7]

Neogastropod new genus B[7]

Austrosphaera bulloides[7,52]

"*Cassidaria*" *mirabilis*[7]

Heteroterma? sp. 1[7]

Heteroterma? sp. 2[7]

Heteroterma sp. [7]

Taioma charcotiana[7]

Cryptorhytis? *phillippiana*[7]

Acteon (*Tenuiacteon*) *antarctihadrum*[53]

Supplementary References

1. Crame, J. A., Francis, J. E., Cantrill, D. J. & Pirrie, D. Maastrichtian stratigraphy of Antarctica. *Cretac. Res.,* **25**, 411–423 (2004).
2. Bowman, V.C. *et al*. The Paleocene of Antarctica: Dinoflagellate cyst biostratigraphy, chronostratigraphy and implications for the palaeo-Pacific margin of Gondwana. *Gondwana Res.* doi:10.1016/j.gr.2015.10.018 (2015).
3. Bowman, V. C., Francis, J. E., Riding, J. B., Hunter, S. J. & Haywood, A. M. A latest Cretaceous to earliest Paleogene dinoflagellate cyst zonation from Antarctica, and implications for phytoprovincialism in the high southern latitudes. *Rev. Palaeobot. Palynol.,* **171,** 40–56 (2012).
4. Bowman, V. C., Francis, J. E. & Riding, J. B. 2013. Late Cretaceous winter sea ice in Antarctica? *Geology* **41**, 1227–1230 (2013).
5. Witts, J. D., Bowman, V. C., Wignall, P. B., Crame, J. A., Francis, J. E. & Newton, R. J. Evolution and extinction of Maastrichtian (Late Cretaceous) cephalopods from the López de Bertodano Formation, Seymour Island, Antarctica. *Palaeogeogr. Palaeoclimatol. Palaeoecol.,* **418**, 193–212 (2015).
6. Bowman, V. C., Francis, J. E., Askin, R. A., Riding, J. B. & Swindles, G. T. Latest Cretaceous–earliest Paleogene vegetation and climate change at the high southern latitudes: palynological evidence from Seymour Island, Antarctic Peninsula. *Palaeogeogr. Palaeoclimatol. Palaeoecol.,* **408**, 26–47 (2014).
7. Crame, J. A., Beu, A. G., Ineson, J. R., Francis, J. E., Whittle, R. J. & Bowman, V. C. The early origins of the Antarctic marine fauna and its evolutionary implications. *PLoS ONE* **9**, e114732 (2014).
8. Marshall, C. R. in: *Quantitative methods in paleobiology.* (eds Alroy, J. and Hunt, G.) **16**, 291–316 (The Pal. Soc., 2010).
9. Tobin, T. S. *et al*. Extinction patterns, δ^{18}O trends, and magnetostratigraphy from a southern high-latitude Cretaceous–Paleogene section: links with Deccan volcanism. *Palaeogeogr. Palaeoclimatol. Palaeoecol.* **350–352**, 180–188 (2012).
10. Gradstein, F. M., Ogg, J. G., Schmitz, M. & Ogg, G. *The Geological Time Scale 2012* Elsevier Science Ltd: Boston, (2012).
11. McArthur, J. M., Thirlwall, M. F., Engkilde, M., Zinsmeister, W. J. & Howarth, R. J. Strontium isotope profiles across K/T boundary sequences in Denmark and Antarctica. *Earth Plan. Sci. Lett.* **160**, 179–*192* (1998).
12. Vonhof, H.B. *et al*. Belemnite-based strontium, carbon, and oxygen isotope stratigraphy of the type area of the Maastrichtian stage. *Neth. J. Geosci.* **90**, 259–270 (2011).

13. Macellari, C.E. Late Campanian-Maastrichtian ammonite fauna from Seymour Island (Antarctic Peninsula). *Mem. Paleontol. Soc*. **18**, pp. 1–55 (1986).
14. Olivero, E. B. Sedimentary cycles, ammonite diversity and palaeoenvironmental changes in the Upper Cretaceous Marambio group, Antarctica*. Cretac. Res.* **34**, 348–366 (2012).
15. Huber, B. T. Upper Campanian–Paleocene foraminfera from the James Ross Island region, Antarctic Peninsula. *Geol. Soc. Am. Mem.* **169,** 163–252 (1988).
16. Harwood, D. M. Upper Cretaceous and lower Paleocene diatom and silicoflagellate biostratigraphy of Seymour Island, eastern Antarctic Peninsula. *Geol. Soc. Am. Mem.* **169,** 55–130 (1988).
17. Zinsmeister, W. J. Discovery of fish mortality horizon at the K–T boundary on Seymour Island: re-evaluation of events at the end of the Cretaceous. *J. Palaeont*. **72**, 556–571 (1998).
18. Macellari, C. E. Stratigraphy, sedimentology, and paleoecology of Upper Cretaceous/Paleocene shelf-deltaic sediments of Seymour Island. *Geol. Soc. Am. Mem.* **169,** 25–53 (1988).
19. Elliot, D. H., Askin, R. A., Kyte, F. T. & Zinsmeister, W. J. Iridium and dinocysts at the Cretaceous–Tertiary boundary on Seymour Island, Antarctica: implications for the K–T event. *Geology* **22**, 675–678 (1994).
20. Meldahl, K. Sampling, species abundance, and the stratigraphic signature of mass extinction: a test using Holocene tidal flat molluscs. *Geology* **18**, 890–893 (1990).
21. Dutton, A., Huber, B. T., Lohmann, K. C. & Zinsmeister, W. J. High-resolution stable isotope profiles of a dimitobelid belemnite: implications for paleodepth habitat and late Maastrichtian climate seasonality *Palaios* **22**, 642–650 (2007).
22. Bond, D. P. G. & Wignall P. B. Pyrite framboid study of marine Permian–Triassic boundary sections: a complex anoxic event and its relationship to contemporaneous mass extinction. *Geol. Soc. Am. Bull.,* **122,** 1265–1279 (2010).
23. Pirrie, D., Crame, J. A., Lomas, S. A. & Riding, J. B. Late Cretaceous stratigraphy of the Admiralty Sound region, James Ross Basin, Antarctica. *Cretac. Res.* **18,** 109–137 (1997).
24. Bowman, V. C., Riding, J. B., Francis, J. E., Crame, J. A. & Hannah, M. The taxonomy and palaeobiogeography of small chorate dinoflagellate cysts from the Late Cretaceous to Quaternary of Antarctica. *Palynology* **37**, 151–169 (2013).
25. Zinsmeister, W. J. & Macellari, C. E. Bivalvia (Mollusca) from Seymour Island, Antarctic Peninsula. *Geol. Soc. Am. Mem.* **169,** 25–53 (1988).
26. Wilckens, O. Die Anneliden, Bivalven und Gastropoden der antarktischen Kreideformation. *Wiss. Ergeb. Schwed. Südpolarexp.*, **3**, 1–132 (1910).

27. Little, C.T.S. *et al*. Late Cretaceous (Maastrichtian) shallow water hydrocarbon seeps from Snow Hill and Seymour Islands, James Ross Basin, Antarctica. *Palaeogeogr. Palaeoclimatol. Palaeoecol.* **418**, 213–228 (2015).
28. Del Valle, R. & Medina, F. A. Nuevos invertebrados fósiles de Cabo Lamb (Isla Vega) y Cabo Morro (Isla James Ross). *Contribuciones del Instituto Antárctico Argentino* **228**, 51–67 (1980).
29. Olivero, E. B., Martinioni, D. & Mussel, F. In: *Geología de la Isla James Ross* (ed. Rinaldi, C. A.), pp. 125–145 (Publicacion Especial del Instituto Antartico Argentino 1992).
30. Askin, R. A., Elliot, D. H., Stilwell, J. D. & Zinsmeister, W. J. Stratigraphy and paleontology of Campanian and Eocene sediments, Cockburn Island, Antarctic Peninsula. *J. S. Am. Ear. Sci.,* **4**, 99–117 (1991).
31. Stilwell, J. D. Geological exploration of Cockburn Island, Antarctic Peninsula. *Polish Polar Res.* **23,** 47–73 (2002).
32. Stilwell, J. D. & Zinsmeister, W. J. Late Cretaceous faunal assemblage of Humps Island collected during the 1986–1987 expedition to the Antarctic Peninsula. *Ant. Jour. US.,* **12**, 9–10 (1987).
33. Stilwell, J. D. & Zinsmeister, W. J. Late Cretaceous fossils from Ula Point, James Ross Island, collected during the 1986–1987 expedition to the Antarctic Peninsula. *Ant. Jour. US.,* **12**, 8–9 (1987).
34. Crame, J. A., McArthur, M. A., Pirrie, D. & Riding, J. B. Strontium isotope correlation of the basal Maastrichtian stage in Antarctica to the European and US biostratigraphic schemes. *J. Geol. Soc. Lond.,* **156**, 957–964 (1999).
35. Cichowolski, M., Ambrosio, A. & Concheyro, A. Nautilids from the upper Cretaceous of the James Ross Basin, Antarctic Peninsula. *Ant. Sci.,* **17**, 267–280 (2005).
36. Bouchet, P. & Rocroi, J-P. Nomenclator of bivalve families. *Malacologia* **52**, 1–184 (2010).
37. Bieler, R. *et al*. Investigating the bivalve tree of life – an exemplar-based approach combining molecular and novel morphological characters. *Invert. Syst.* **28**, 32–115 (2014).
38. Bouchet, P. & Rocroi, J-P. Classification and nomenclator of gastropod families. *Malacologia* **47**, 1–397 (2005).
39. Ponder, W. F. & Limberg, D. R. *Phylogeny and evolution of the Mollusca*. 469 p. (University of California Press, 2008).
40. Stilwell, J. D., Zinsmeister, W. J. & Oleinik, A. E. Early Paleocene mollusks of Antarctica: systematics, paleoecology and paleogeographic significance. *Bull. Am. Paleontol.* **367**, 1–89 (2004).

41. Beu, A. G. Before the ice: biogeography of Antarctic Paleogene molluscan faunas. *Palaeogeogr. Palaeoclimatol. Palaeoecol.,* **284**, 191–226 (2009).
42. Whittle, R. J., Linse, K. & Griffiths, H. J. The fossil record of *Limopsis* (Bivalvia: Limopsidae) in Antarctica and the southern high latitudes. *Palaeontology* **54**, 935–952 (2011).
43. Zinsmeister, W. J. Three new species of *Pulvinites* (Mollusca: Bivalvia) from Seymour Island (Antarctic Peninsula) and southern California. *J. Paleontol.,* **52**, 565–569 (1978).
44. Machalski, M. Redescription of a Danian oyster *Pycnodonte simile* (Pusch, 1837) from Poland. *Act. Pal. Pol.* **33**, 73–83 (1988).
45. Wilmsen, M. & Voigt, T. The middle-upper Cenomanian of Zilly (Sachsen-Anhalt, northern Germany) with remarks on the *Pycnodonte* event. *Act. Geol. Pol.* **56**, 17–31 (2006).
46. Waller, T. R. Phylogeny of families in the Pectinoidea (Mollusca: Bivalvia) importance of the fossil record. *Zool. J. Linn. Soc.* **148**, 313–342 (2006).
47. D'Hondt A. Systematic revision of *Entolium*, *Propeamussium* (Amusiidae) and *Syncyclonema* (Pectinidae, Bivalvia, Mollusca) of the European Boreal Cretaceous. *Bull. Inst*. *Roy*. *Sci. Nat. Belg*., **47**, 1–95 (1971).
48. Stilwell, J. D. Late Cretaceous Mollusca from the Chatham Islands, New Zealand. *Alcheringa* **22**, 29–85 (1998).
49. Harasewych, M. G., Oleinik, A. & Zinsmeister, W. J. The Cretaceous and Paleocene Pleurotomariid (Gastropoda: Vetigastropoda) fauna of Seymour Island, Antarctica. *J. Paleontol.,* **83**, 750–766 (2009).
50. Ferrari, S. M., Kaim, A. & Damborenea, S. E. The genera *Calliotropis* Seguenza and *Ambercyclus* n. gen. (Vetigastropoda, Eucyclidae) from the early Jurassic of Argentina. *J. Paleontol.,* **88**, 1174–1188 (2014).
51. Zinsmeister, W. J. & Griffin, M. Late Cretaceous and Tertiary aporrhaid gastropods from southern rim of the Pacific Ocean. *J. Paleontol.,* **69**, 692–702 (1995).
52. Oleinik, A. E. & Zinsmeister, W. J. Paleocene diversification of bucciniform gastropods on Seymour Island, Antarctica. *J. Paleontol.,* **70**, 923–934 (1996).
53. Stilwell, J. D. & Zinsmeister, W. J. A new, large, Acteonid gastropod (Mollusca) from the latest Cretaceous of Antarctica. *J. Paleontol.,* **76**, 1102–1105 (2002).
